# Cyclophosphamide in pulmonary alveolar hemorrhage due to leptospirosis

**DOI:** 10.4103/0972-5229.56053

**Published:** 2009

**Authors:** Samir V. Trivedi, Ashwin H. Vasava, Tinkal C. Patel, Lovleen C. Bhatia

**Affiliations:** **From:** Department of Medicine, Government Medical College, Surat, India

**Keywords:** Cyclophosphamide, leptospirosis, pulmonary alveolar hemorrhage

## Abstract

**Background and Aims:**

Severe pulmonary involvement in leptospirosis carries high mortality rates. It is the most common cause of death due to leptospirosis in many parts of India and the world. Exacerbated immune response of the host plays an important role in its pathogenesis. Hence, immunosuppressive drugs could be useful in its treatment. Glucocorticosteroids have been found to be useful in several studies. Cyclophosphamide, an immunosuppressive agent, has been found to be useful in a majority of pulmonary alveolar hemorrhages due to non leptospiral causes. This study was carried out to study the effects of cyclophosphamide in patients with leptospiral pulmonary alveolar hemorrhage.

**Method:**

A total of 65 patients with confirmed leptospirosis with severe pulmonary involvement admitted to a tertiary care center in south Gujarat were included in the study. All of the patients were treated with injection crystalline penicillin, methyl prednisolone pulse therapy, and non invasive mechanical ventilation. A total of 33 patients were given parenteral cyclophosphamide 60 mg/kg body weight stat on diagnosis. Their outcomes were compared with the remaining 32 patients who had not been given this drug. Survival was considered the main outcome indicator.

**Results:**

Out of the 33 patients treated with cyclophosphamide, 22 (66.7%) survived, while in the control group out of 32 patients, three (9.4%) survived. On statistical analysis, the odds ratio was 19.33 (4.22–102.13) and the *P*-value was <0.001. Leucopenia (78.78%) and alopecia (18.75%) were the main side effects noted. No mortality was noted due to these side effects.

**Conclusion:**

Cyclophosphamide improves survival in cases of severe pulmonary alveolar hemorrhage due to leptospirosis. Statistically, the improvement is highly significant.

## Introduction

Pulmonary involvement in leptospirosis has been reported from all over the world.[1] In India, it has also been documented from all the places where leptospirosis cases occur. In fact, it is the most common cause of death due to leptospirosis in several parts of India.[2,3] The basic pathology in pulmonary involvement is alveolar hemorrhage.[4] Immune causes are responsible for the majority of non leptospiral pulmonary alveolar hemorrhages.[5] Vasculitis is one of the more important of these immune causes. They are all treated with prednisolone and cyclophosphamide.[5]

Immune mechanisms have been implicated in the pathogenesis of alveolar hemorrhage due to leptospirosis as well.[1] Leptospirosis is an infectious vasculitis.[6] There are reports of the successful use of glucocorticosteroids in leptospiral alveolar hemorrhage.[7–11] But, there are reports that cases of leptospirosis are becoming more severe all over the world.[1] In Gujarat, we have noted an increase in the severity of leptospirosis with decreasing response to glucocorticosteroids. (This aspect is elaborated further in the Discussion section.) Immune mechanisms have been implicated for the increasing severity of leptospirosis. Cyclophosphamide has been an integral part of the treatment regimen used to treat non leptospiral causes of pulmonary hemorrhage.[5] Hence, we decided to evaluate its role in pulmonary alveolar hemorrhage due to leptospirosis. Results of the study are presented in this paper.

## Materials and Methods

### Patient selection

At the outset, approval by the Human Research Ethics Committee of the college was obtained.

The study was carried out at a tertiary care center in south Gujarat where severe leptospirosis patients from three districts are referred. The study was carried out between July 2007 and October 2007. This is the peak season for leptospirosis in south Gujarat because of the monsoon season. Suspected patients were subjected to serological tests for leptospirosis and polymerase chain reaction (PCR). The serological tests included dipstick tests for lepto antibodies, IgM enzyme linked immunosorbent essay (ELISA), and microscopic agglutination test (MAT). The World Health Organization (WHO) International Leptospirosis Society (ILS) guidelines were followed for the diagnosis of leptospirosis.[12] Besides a clinical examination, certain investigations were carried out in patients with confirmed leptospirosis. They included complete blood counts, renal function tests, hepatic function tests, and arterial blood gas analysis. Tests for the diagnosis of hepatitis B and C, dengue (IgM), and malaria (peripheral smear and rapid test) were also carried out. A chest X-ray, electrocardiogram (ECG), and abdominal ultrasound were also done. The presence of respiratory symptoms or signs (breathlessness, tachypnoea, cough, or hemoptysis) and/or a chest X-ray showing infiltrates, was considered as pulmonary involvement. An acute lung injury (ALI) score, developed by Murray *et al*., was used to determine the severity of pulmonary involvement.[13] Patients having an ALI score ≥2.5 were considered to have severe pulmonary involvement and were included in the study. Other causes of opacities in the lungs, like tuberculosis, pneumonia, etc. were ruled out by appropriate clinical and laboratory methods. Out of 437 confirmed cases of leptospirosis, 167 (38.7%) had pulmonary involvement. A total of 65 had an ALI score ≥ 2.5 and were included in the study.

### Treatment protocol

All patients were given the following treatment: (1) Inj. Crystalline penicillin 2 × 10^6^ units intravenously 6 hourly, (2) Inj. Methyl prednisolone 1 gram intravenously daily for 3 days, followed by oral prednisolone 1 mg per kg of body weight for 7 days, (3) Non invasive mechanical ventilation) was used where necessary. There are studies to indicate that non invasive ventilation is an affordable alternative to treat patients with severe acute lung injury.[9,14] Blood component therapy was given as and when indicated.

Out of the first 32 patients who had been given this treatment, only three survived. In view of the poor outcome in the initial 32 patients, and considering the fact that immune mechanisms are important in its pathogenesis, the next 33 patients were given a trial of cyclophosphamide. They were all given injection cyclophosphamide 60 mg/kg body weight intravenously immediately upon the diagnosis of severe pulmonary hemorrhage. Mesna (2-MercaptoEthane Sulfonate sodium) was also given to prevent hemorrhagic cystitis.

### Monitoring and outcome

Chest X-rays were taken at regular intervals. Blood total counts were taken daily for 21 days. Platelet counts, liver function tests, and renal function tests were performed at regular intervals. All patients who survived were observed in the hospital for 21 days or until the total counts returned to normal, whichever was longer. Survival was taken as the outcome indicator.

### Statistical analysis

Survival rates were compared between the two groups: Group I - patients who were given cyclophosphamide and Group II - patients who were not given cyclophosphamide. Odds ratio and p-value were calculated to assess the statistical significance of the study. In view of the life-threatening nature of the disease, a randomized trial was not possible.

## Results

A total of 33 patients were enrolled for treatment with cyclophosphamide (Group I). Of these patients, 26 were males. The mean age of the patients was 33 years old. The youngest patient was 17 years old and the oldest patient was 60 years old. The group of patients who had not been given cyclophosphamide had similar demographic characteristics [Table 1]. All were farm laborers by occupation working in either paddy or sugarcane fields which are water logged.

**Table 1:** Demographic data of the two groups

		Group I (Cyclophosphamide given) N=33	Group II (Cyclophosphamide not given) N=32	P-Value
Age group	15-45 yrs (n=55)	29(52.7%) patients	26(47.%) patients	0.56
	>45 yrs. (n=10)	4(40%) patients	6 patients (60%)	0.37
Sex	Male (n=47)	26(55.3%)	21 (44.7%)	0.3
	Female (n=11)	07(63.6)	11 (36.4%)	0.2

Table 2 shows the symptomatology of the two groups. Fever, myalgias, and headache were the most common symptoms. Although all the patients had pulmonary hemorrhage, cough and hemoptysis were present in only a small percentage of patients. As a matter of fact, tachypnoea was the best clinical indicator of pulmonary involvement. Table 3 shows organ involvements in the two groups. Eleven patients were given platelet transfusions in the treated group while 14 patients were given platelet transfusions in the control group. None of the patients required dialysis. As already mentioned, all patients in both the groups required non invasive ventilation, BiPAP, with positive end expiratory pressure {PEEP}. In the patients who survived, the median duration for which mechanical ventilation was required was 122 hours in Group I and 128 hours in Group II. The mean duration was 124.8 hours SD ± 30.31 and 123.3 hours SD ± 9.8 in Groups I and II, respectively.

**Table 2:** Symtomatology in the two groups

	Group I Cyclophosphamide given (n=33)	Group II Cyclophosphamide not given (n=32)	P-value
Fever	33 (100)	32 (100)	>0.05
Myalgia	31 (93.9)	30 (93.75)	>0.05
Headache	25 (75.75)	27 (84.37)	>0.05
Cough	16 (48.48)	15 (46.87)	>0.05
Hemoptysis	8 (24.24)	8 (25)	>0.05
Tachypnoea	29 (87.8)	28 (87.5)	>0.05

Figures in parentheses are in percentage

**Table 3:** Incidence of organ involvement in the two groups

	Group I Cyclophosphamide given (n=33)	Group II Cyclophosphamide not given (n=32)	P-value
Lungs	33 (100)	32 (100)	>0.05
Kidney	26 (81.25)	27 (81.8)	>0.05
Liver	24 (75)	24 (72)	>0.05
Thrombocytopenia	25 (75.75)	23 (72.8)	>0.05

Figures in parentheses are in percentage

Leucopenia and alopecia were the main side effects noted. A total of 26 patients (78.78%) developed leucopenia. The lowest total white blood cell (WBC) count noted was 400. The median duration after which maximum leucopenia was noted was 10 days while the mean duration was 10.05 SD + 0.99. All patients were given colony stimulating factor and broad spectrum antibiotics. The median time taken for the total WBC count to return to normal was 17 days while the mean was 17.33 SD ± 0.84. Three patients developed a secondary infection. Two of them developed pneumonia while one patient developed a urinary tract infection. All of them had complete recovery. None of the patients died due to leucopenia or any other side effects. Six (18.75%) patients developed alopecia. None of the patients developed hemorrhagic cystitis, obviously due to the simultaneous administration of Mesna (2-MercaptoEthane Sulfonate sodium). Platelet counts improved in patients who survived. The average duration for the clearance of opacities from the lungs was seven days.

**Figure 1:**
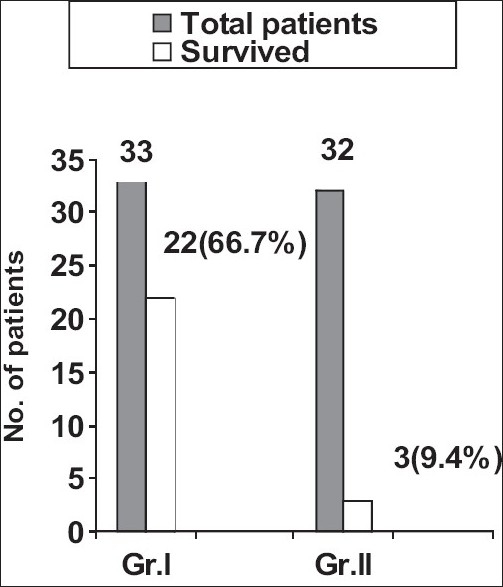
Response to cyclophosphamide in severe alveolar hemorrhage due to leptospirosis; GrI=cyclophosphamide given; Group II = cyclophosphamide not given

**Figure 2:**
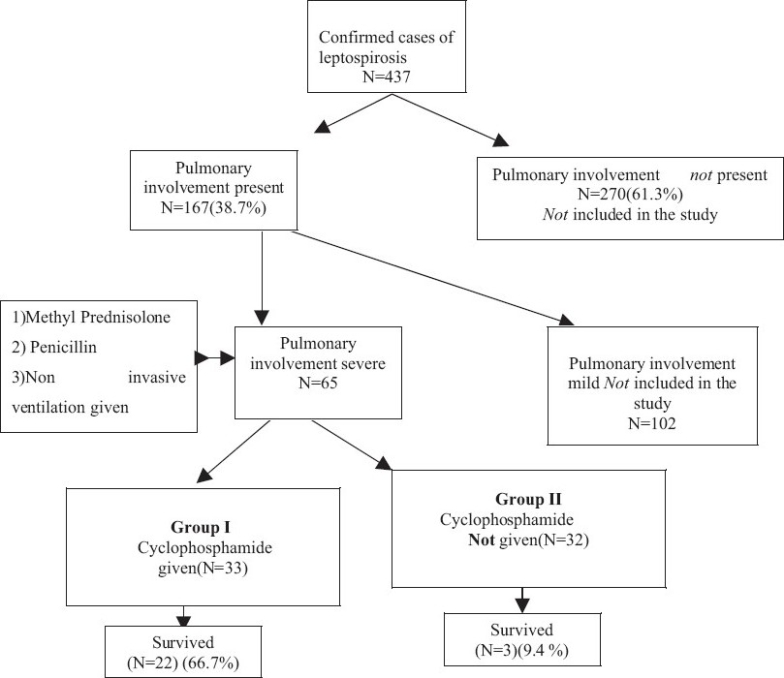
Flowchart showing the protocol and outcome of the study

In the group of patients who had been given cyclophosphamide, 22 out of 33 patients survived (66.7%) while in Group II three out of 32 patients survived (9.4%) [Figures 1 and 2].

### Statistical analysis

The odds ratio was 19.33 (4.22-102.1) indicating that survival was 19.33 times more among those who were given cyclophosphamide as compared with those who were not treated with the drug. The *P*-value was < 0.001 indicating that the association between survival and treatment with cyclophosphamide was statistically highly significant.

## Discussion

There was a male predominance in this study. This is explained by the fact that all the patients are farm laborers and there are more males in this profession. Predominance of younger patients can also be explained in the same way.

Mainly, two mechanisms have been proposed for the pathogenesis of lung injury in leptospirosis: a toxin mediated mechanism and/or exacerbated immune responses of the host.[1] There is evidence to suggest that immune mechanisms play a key role in the pathogenesis of pulmonary alveolar hemorrhage due to leptospirosis. Some of the more important mechanisms are as follows:

- Leptospirosis is an infectious vasculitis and immune mechanisms play an important role in the pathogenesis of all vasculitis.[6]
- The majority of pulmonary alveolar hemorrhages due to non leptospiral causes occur due to immune mechanisms. They include anti-basement membrane antibody disease (Goodpasture's syndrome), vasculitis (Wegener's granulomatosis, etc.), collagen vascular disease, etc. Vasulitis is one of the more important of these causes.[5]
- There are two phases in the clinical manifestations of leptospirosis: septicemic phase and an immune phase.[6] A majority of complications occur in the immune phase when the organisms disappear from the bloodstream and the antibodies appear. This phase is called the immune phase obviously because of the underlying immune pathogenesis. Immune mediated disease has been proposed as one factor influencing the severity of disease.[15]
- IgG, IgA, and C3 deposition has been demonstrated along the alveolar basement membrane of infected guinea pigs in a similar pattern to that seen in Goodpasture's syndrome.[16] In view of presence of antibodies and complement and the paucity of spirochetes in lung tissue, authors have postulated that the infection might have precipitated an immune process that led to pulmonary hemorrhage through damage to the alveolar septa.
- A patient of leptospirosis who died of respiratory failure had similar findings.[17]
- In a Brazilian study, Abdulkader, *et al*. found that patients with higher IgG titers (>400) had more severe pulmonary hemorrhage and renal injury, when compared with patients with lower IgG titer (400).[18] It indicates that the severity of Weil's disease may be proportional to the humoral immune response to leptospires.
- Glucocorticosteroids, which are known to have immunosuppressive effects, have been found to be useful in pulmonary hemorrhage due to leptospirosis in several studies.[7–11]
- Plasma exchange has been found to be useful in leptospirosis.[19,20] Plasma exchange is useful mainly in conditions where immune mechanisms play an important role.
- Meaudre,*et al.* found intravenous immunoglobulin to be useful in pulmonary leptospirosis.[21] Immunoglobulins are useful mainly in conditions where immune mechanisms play an important role.
- Werts, *et al*. have suggested that leptospiral infection triggers innate immunity by LPS- activating macrophages.[22]

There are reports from various parts of the world that the pattern of organ involvement and severity of leptospirosis are changing. There is increasing involvement of lungs and the disease severity is also increasing.[1] Daher, *et al*. have reported from Brazil that clinical patterns of Weil's disease have changed; more severe cases are now being detected mainly with pulmonary involvement.[23] Abdulkader, *et al*. suggested that those changes could result from an intense immune response consequent to previous leptospira infection as it occurs in dengue hemorrhagic fever.[17,24] Thus, patients who have reinfection are likely to have more severe disease.

**Figure 3:**
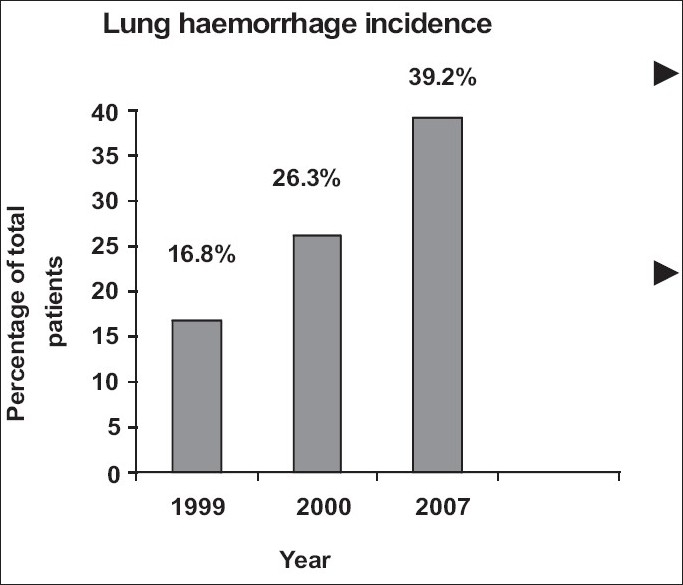
Increasing incidence of pulmonary hemorrhage in patients of leptospirosis in south Gujarat

The changing pattern of leptospirosis has been observed by us in south Gujarat as well. We reported 16.8% and 26.3% incidences of pulmonary involvement in studies carried out in 1999 and 2000, respectively.[8,2] The present incidence of 38.7% in 2007 is much higher, indicating an increasing involvement of the lungs [Figure 3]. More alarming than the increasing incidence of pulmonary involvement is the increasing severity of the disease. We had found glucocorticosteroids to be quite effective in our study conducted in 1999.[8] But in the present study, the response to glucocorticosteroids was very poor. The explanation put forward by Abdulkader, *et al*. is applicable to our observations as well. South Gujarat has been witnessing leptospirosis since 1997.[2] In the initial years, we received patients who were infected for the first time, hence the disease was mild. But as the disease became endemic, patients started to have reinfection. The second infection is more severe in a manner similar to dengue.[24]

As leptospirosis is an infection, antibiotics have been used for a long time as treatment. In the Cochrane database review, Guidugli, *et al*. looked at the role of antibiotics in the treatment of leptospirosis.[25] The review of trials found there was not enough evidence to show the benefit or safety of antibiotics for leptospirosis. There is no evidence of resistance to the traditionally used antibiotics such as penicillin.[1] Thus, changing the antibiotics or increasing their dosages will not help in decreasing the mortality rates.

Thus, we have gathered the following evidence so far:

- Immune mechanisms play an important role in the pathogenesis of the disease.
- All immune, non leptospiral pulmonary hemorrhages are treated with glucocorticosteroids and cyclophosphamide.
- Glucocorticosteroids have been found to be useful but as the disease is increasing in severity, their efficacy is decreasing.
- Antibiotics do not have a definite, proven role and leptospira is still sensitive to the currently used antibiotics.

In light of the above evidence, it is rational to assume that cyclophosphamide could be effective in this condition. Results of this study validate our assumption. Cyclophosphamide is an alkylating agent.[26] It acts by interfering with DNA integrity and function in rapidly proliferating tissues. It has been part of the standard treatment protocol for the treatment of non leptospiral pulmonary hemorrhage for a very long time.[5] Various doses of cyclophosphamide have been described. We decided to evaluate a very high dose because these patients succumb to the disease in a very short time - sometimes even less than 24 hours.[27] Obviously, in such situations low dose, long-term therapy has no value. The main concern with such a high dose is leucopenia and secondary infection. A total of 26 patients (78.78%) developed leucopenia, albeit reversible, in this study.

Abdulkader *et al*. have carried out a serological study to suggest an exacerbated immune response of the host as an important mechanism of pathogenesis. We have postulated that the same is operative in South Gujarat but serological studies should be carried out to validate our hypothesis.

We conclude that cyclophosphamide therapy is very useful in the treatment of pulmonary alveolar hemorrhage due to leptospirosis. It provides a new tool for the treatment of this dreaded disease. At the same time, it also provides added supportive evidence for the hypothesis that immune mechanisms play a pivotal role in the pathogenesis of this disease.
